# Elevated Circulating Interleukin-27 in Patients with Coronary Artery Disease Is Associated with Dendritic Cells, Oxidized Low-Density Lipoprotein, and Severity of Coronary Artery Stenosis

**DOI:** 10.1155/2012/506283

**Published:** 2012-07-13

**Authors:** Wen Jin, Yiqiao Zhao, Wen Yan, Longxing Cao, Weiwei Zhang, Ming Wang, Ting Zhang, Qiang Fu, Zhiliang Li

**Affiliations:** ^1^Cardiovascular Department, Guangdong No.2 Provincial People's Hospital, Guangdong 510317, China; ^2^Oncology Department, Guangdong No.2 Provincial People's Hospital, Guangdong 510317, China; ^3^Cardiovascular Department, Zhujiang Hospital, Southern Medical University, Guangdong Province, Guangdong 510282, China; ^4^Traditional Chinese Medicine Department, Zhujiang Hospital, Southern Medical University, Guangdong 510282, China

## Abstract

Coronary artery disease (CAD) is an immune-mediated chronic inflammatory disease mainly caused by atherosclerosis. The aims of this study were to investigate the role of interleukin-27 (IL-27) in patients with CAD and the severity of coronary artery lesions, which was evaluated by Gensini score and to investigate the biosynthesis of IL-27 and oxidized low-density lipoprotein (ox-LDL) *in vitro* using monocyte-derived dendritic cells (DCs). To this aim, plasma levels of IL-27, ox-LDL, and Gensini score were analyzed in patients with CAD (*n* = 136) and normal subjects (controls, *n* = 29). IL-27 concentration of the supernatant and the mRNA expression levels of p28 and ebi3, subunits of IL-27, from cultured immature DCs incubated with different concentrations of ox-LDL for 24 h were also analyzed. We found that circulating IL-27 levels were significantly elevated in patients with CAD than in controls (*P* < 0.01), and positively correlated to ox-LDL and Gensini score. ox-LDL dose-dependently upregulated expression of both IL-27 protein and IL-27 (p28 and EBI3) mRNA *in vitro,* indicating that ox-LDL can stimulate DCs to produce IL-27. These results demonstrate that IL-27 might regulate the network of immunity and inflammation in the pathogenesis of atherosclerosis.

## 1. Introduction

Coronary artery disease (CAD) remains the leading cause of death worldwide despite advances in prevention and treatment [1]. Additional insight into the mechanisms of the development of atherosclerosis and the underlying cause of CAD is needed to improve treatment outcomes of these patients. Particularly the contribution of immune responses with cumulating evidence suggests that atherosclerosis is a chronic immune-inflammatory disease [2].

Dendritic cells (DCs), most efficient antigen-presenting cells in the immune system, have emerged as key players in initiating and regulating adaptive immune responses [3–5]. Current research has recognized dendritic cells as key initiators and regulators of immune processes in atherosclerosis [6, 7]. DCs can modulate immune responses by a variety of mechanisms in the pathogenesis of atherosclerosis [8–10]. This includes expression of  T cell costimulatory and regulatory molecules, as well as the production of chemokines and cytokines. The secretion of interleukin-(IL-) 12, IL-10, and other cytokines by DCs plays a critical role in polarizing naive T cells into Th1, Th2, T regulatory cells (Treg), or Th17 cells, which are known to drive or dampen inflammatory processes in atherosclerosis [11].

Recently, IL-27, mainly produced by DCs, has been identified as cytokines belonging to the IL-12 family [12]. IL-27 is a heterodimeric cytokine composed of EBI-3, a p40-related molecule [13], and p28, a p35-related molecule [14]. IL-27 receptor complex comprises IL-27R (also called WSX-1 or T-cell cytokine receptor) and glycoprotein 130 (gp130) [15]. IL-27R and GP130 are coexpressed on different cell types, such as monocytes, macrophages, DCs, mast cells, NK cells, endothelial cells, and T and B lymphocytes [14, 16–18] whilst IL-27R is the only known receptor for IL-27 [14]. These may be the molecular basis for the wide-ranging immunomodulatory function of IL-27. Available evidence suggests that IL-27, unlike other members of this cytokine family, has dual function: one as an initiator and the other as an attenuator of immune/inflammatory responses [19].

Given the main effect of IL-27 is the regulation of the innate and adaptive immunity, it is most likely to be involved in atherosclerosis. However, little information is known about the role of IL-27 in atherosclerosis. The aim of this study was therefore to assess the levels of IL-27 in plasma of patients with CAD and produced by DCs *in vitro* stimulated by oxidized low-density lipoprotein (ox-LDL). We have demonstrated that (1) the circulating levels of IL-27 are elevated in patients with CAD, particularly in ACS, and correlated with ox-LDL and Gensini score; (2) IL-27 can be secreted from human monocyte-derived DCs in response to *in vitro* stimulation with ox-LDL, indicating an important role for IL-27 in the pathogenesis of atherosclerosis.

## 2. Methods and Materials

### 2.1. Study Protocol

The study protocol conforms to the principles of the Declaration of Helsinki and was performed with approval of the Ethics Committee of South Medical University. Subjects were selected from individuals who underwent coronary angiography to investigate ischemic heart disease based on clinical indications (typical and atypical chest discomfort) from November 2008 to December 2009. All subjects are Han Chinese. All subjects gave informed consent, both verbally and in writing, for participation in the study, and underwent coronary artery angiography at Zhujiang Hospital of South Medical University before entering the study. Routine blood analyses were performed in our hospital clinical laboratory.

In total, 165 subjects (113 men and 52 women, age range from 32 to 84 years with mean age of 62.16 ± 9.78 years) were studied. Patients diagnosed with coronary heart disease had to have had at least one severe stenosis (>50%) in a major coronary artery, as determined by diagnostic coronary angiography.

The patients were divided into four study groups. The first group included patients with stable angina pectoris (SAP) that had a long-term, stable effort angina that had lasted at least three months and a positive exercise test. The second group included patients with unstable angina pectoris (UAP), as defined by as either angina with a progressive crescendo pattern or angina that occurred at rest without a recent myocardial infarction. In those patients, transient ST-T segment depression and T-wave inversion were often present, but no significant elevation of cardiac enzymes was detected. Patients with acute myocardial infarction (AMI) had typical angina associated with ST-segment elevations in electrocardiogram and/or elevated plasma troponin-I. The fourth group, controls, consisted of patients with normal coronary artery angiographies. In total, we recruited 29 controls, 30 patients with SAP, 56 patients with UAP, and 50 patients with AMI.

Exclusion criteria were established for patients with autoimmune, neoplastic, liver, hematological, or renal diseases, recent surgery or recent trauma, and/or chronic inflammatory conditions. In addition, patients with valvular heart disease, nonischemic cardiomyopathy, and/or cerebrovascular disease were also excluded. Also, patients who took medications, such as immunosuppressive agents, statins, angiotensin-converting enzyme inhibitors, and angiotensin receptor blockers (before enrollment) were also excluded.

### 2.2. Detecting Plasma Concentrations of IL-27 or Oxidized Low-Density Lipoprotein (ox-LDL)

Prior to coronary angiography, blood samples for determination of IL-27 or ox-LDL were collected in EDTA-citrated tubes, centrifuged, and the isolated plasma was stored at −80°C until analysis within 13 months from sampling. Concentrations of IL-27 (Biolegend, San Diego, USA) or ox-LDL (Calbiochem, San Diego, USA) in plasma were determined at the same time using enzyme-linked immunosorbent assay (ELISA) kits according to the manufacturer's instructions. Concentrations were calculated by regression analysis of a standard curve.

### 2.3. Determining the Severity of Coronary Artery Stenosis by Gensini Score

Gensini score was used to evaluate the severity of coronary artery stenosis from coronary angiography [20, 21]. The procedures were carried out as follows. Selective coronary angiography was conducted by two experienced interventional cardiologists blinded with patient's clinical characteristics and biochemical results. The severity of coronary artery stenosis was assessed by quantitative coronary angiography. Gensini score was used to assess the severity of coronary artery stenosis. According to the degree of luminal narrowing and its location, the Gensini score was calculated by assigning a value to each coronary stenosis. Details of Gensini score are as follows: 1–25%, 26–50%, 51–75%, 76–90%, 91–99%, and 100% of coronary luminal narrowing were given scores of 1, 2, 4, 8, 16, and 32, respectively, which were then multiplied by a factor that represents the importance of the lesion's position in the coronary arterial system: 5 for the left main coronary artery, 2.5 for the proximal segment of the left anterior descending coronary artery (LAD) or the circumflex artery (LCX), 1.5 for mid-segment of LAD, 1 for distal segment of the LAD or the posterolateral artery or the obtuse marginal artery or right coronary artery, and 0.5 for all others.

### 2.4. Isolation of Human Monocyte-Derived DCs and Culture

 Peripheral blood mononuclear cells (PBMC) were isolated from buffy coats of healthy donors using Ficoll gradient. Monocytes were isolated and cultured (1.0 × 10^6^/mL) as previously described using CellGro DC Medium (CellGenix, Freiburg, Germany) supplemented with 50 ng/mL GM-CSF and 50 ng/mL IL-4 (Peprotech, New Jersey, USA) for 6 days to obtain immature DCs. For differentiation into mature DCs, immature DCs cells were resuspended at 6 × 10^5^/mL and further cultured with different concentrations of oxidized low-density lipoprotein in DC medium supplemented with 50 ng/mL GM-CSF and 50 ng/mL IL-4 for 24 h. The supernatants of DC cultures (24 h incubation with different concentrations of ox-LDL) were harvested and the concentrations of IL-27 were analyzed using ELISA kits according to the manufacturer's instructions. DCs were used for the following experiments.

### 2.5. Phenotypic Analysis of DC by Flow Cytometry

DCs were resuspended at 1.0 × 10^5^ cells in 100 *μ*L of PBS and incubated for 30 min at 4°C with different fluorescein isothiocyanate- (FITC-) conjugated monoclone antibodies (mAbs) or appropriate isotype controls. After three washes in cold phosphate-buffered saline (PBS) supplemented with 0.5% of bovine serum albumin (BSA), cells were fixed with 2% paraformaldehyde in PBS. The following mAbs were used: FITC-anti-CD83, FITC-anti-CD86, and FITC-anti-HLA-DR (all from BD Biosciences, USA). Phenotypic data were acquired using a FACScan system (BD Biosciences, USA) and analyzed with Cell Quest software.

### 2.6. Using Quantitative Real-Time PCR (qRT-PCR) to Determine the mRNA Expression Levels of p28 and ebi3 in DCs

Immature DCs were stimulated with ox-LDL (100 mg/mL) for 0, 8 h, 16 h, 24 h, and then collected. Total RNA was extracted from the collected cells using an RNeasy mini kit (Qiagen). Firstly, reverse transcription (RT) reactions were carried out as follows. 1 *μ*g aliquots of total RNA were mixed with 1 *μ*L of oligo (dT) primers (0.5 mg/mL), 1 *μ*L of 10 mM dNTPs, and double-distilled H_2_O to equalize volumes of all samples at 12 *μ*L. The mixture was heated at 65°C for 5 min, quenched on ice, and spun down briefly, and 8 *μ*L of master mix was added. The RT master mix consisted of 4 *μ*L of 5 × first strand buffer (Invitrogen), 2 *μ*L of 0.1 M DTT, 1 *μ*L of RNase inhibitor (40 units/*μ*L; Invitrogen), and 1 *μ*L of Superscript II (200 *μ*L/*μ*L; Invitrogen). The reaction was incubated at 42°C for 60 min and then at 70°C for 15 min, followed by a 4°C soak. To each sample (in a 20 *μ*L total volume) 80 *μ*L of double-distilled H_2_O was added. 5 *μ*L of diluted cDNA was used for each PCR of 25 *μ*L volume. The following primers were used: for PCR amplification of the human p28 cDNA, GCT GGC GGC TCA GCC TGT TG (Forward) and AGC AGC TTC CTG GCG AGA TG (Reverse); for human ebi3 cDNA, CAT AAC AGA GCA CAT CAT CAA GCC (Forward) and GCT TGT AAC GGA TCC AGT ACT TCA (Reverse); for human *β*-actin cDNA, TGG CAC CCA GCA CAA TGA A (Forward) and CTA AGT CAT AGT CCG CCT AGA AGC A (Reverse).

After that, qRT-PCR was used to determine the levels of mRNA expression. Briefly, cDNA samples converted from 1 *μ*g of total RNA were diluted and studied at several concentrations. Diluted cDNA was mixed with a pair of primers (10 *μ*M) targeting human p28, ebi3, or *β*-actin cDNA sequences as described above and with SYBR Green PCR master mix (Applied Biosystem, CA) in a 15 *μ*L volume. PCR cycling was as follows: 2 min at 50°C, 10 min at 95°C for 1 cycle, followed by 40 cycles for 15 s at 95°C, 1 min at 60°C. Dissociation curves were routinely used to demonstrate that there is only one product produced for each reaction.

### 2.7. Statistical Analysis

Statistical analysis was performed using SPSS software, version 13.0 (SPSS Inc., Chicago, USA). Continuous variables were expressed as mean ± SD, and categories were expressed as percentages. Data distribution was assessed by the Shapiro-Wilk's test. Variables were compared by One-Way ANOVA or *χ* ^2^ test. Proportions were compared by *χ* ^2^ test. Correlation coefficients were assessed by Pearson's product-moment correlation. A *P* value of less than 0.05 was considered statistically significant.

## 3. Results

### 3.1. Clinical Characteristics of Patient Cohort

The clinical characteristics and laboratory data of subjects are summarized in Table 1. The age, gender ratio, risk factors, medications, the levels of serum total cholesterol, triglycerides, creatinine, and Leucocyte counts were similar in overall groups. The levels of serum low-density lipoprotein cholesterol are higher in AMI, UAP, and SAP than in control (*P* < 0.05). The levels of serum uric acid are higher in AMI than in UAP, SAP, and control (*P* < 0.05). Differences in the levels of serum cardiac troponin I were observed among all groups (*P* < 0.001).

### 3.2. Circulating IL-27, ox-LDL, and Gensini Score Levels in CAD

The levels of IL-27 and ox-LDL in serum samples from patients with CAD and controls were assessed by ELISA. As shown in Table 2, circulating IL-27 levels were significantly elevated in patients with CAD than in controls (*P* < 0.01). Furthermore, Circulating IL-27 levels were higher in patients with AMI and UAP than in patients with SAP (*P* < 0.01). ox-LDL levels in patients with AMI were significantly higher than in patients with UAP (*P* < 0.05), or SAP (*P* < 0.01), or in controls (*P* < 0.01). The levels of ox-LDL in patients with UAP and SAP were significantly higher than those in controls (*P* < 0.05). The Gensini score was significantly higher in patients with AMI than in patients with UAP (*P* < 0.05), or SAP (*P* < 0.01), or in controls (*P* < 0.01).

### 3.3. Correlation among Circulating IL-27, ox-LDL, and Gensini Score

**Figure 1:**
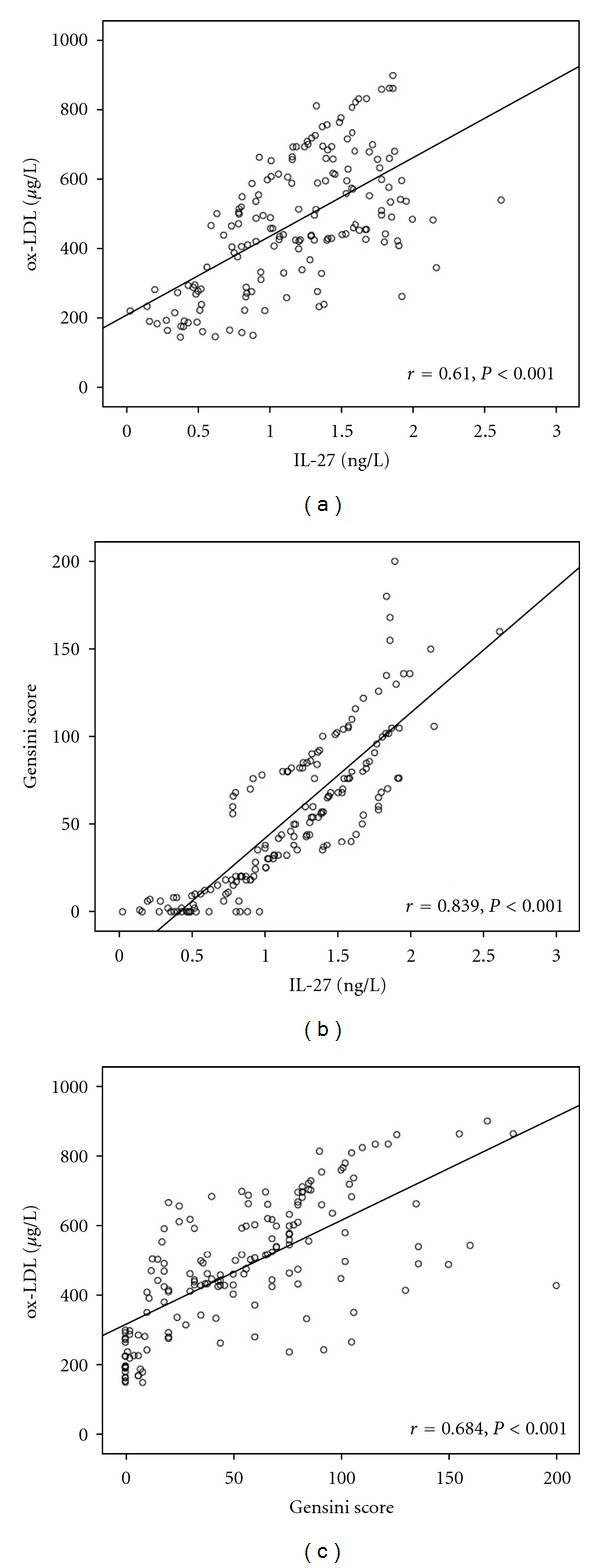
Correlation analysis of circulating levels of IL-27, ox-LDL, and Gensini score in the overall population. Circulating levels of IL-27 were positively correlated with ox-LDL (a), Gensini score (b); Circulating levels of ox-LDL were positively correlated with Gensini score (c). *r*: correlation coefficient.

As shown in Figure 1, circulating IL-27 levels were significantly positively correlated to ox-LDL and Gensini score (*P* < 0.01). Similarly, the levels of ox-LDL were positively correlated with Genisi score (*P* < 0.01). The results indicated that increased IL-27 levels correlated with the severity of coronary artery stenosis.

### 3.4. IL-27 Secretion by Human Monocyte-Derived DCs in Response to *In Vitro* Stimulation with Oxidized Low-Density Lipoprotein

**Figure 2:**
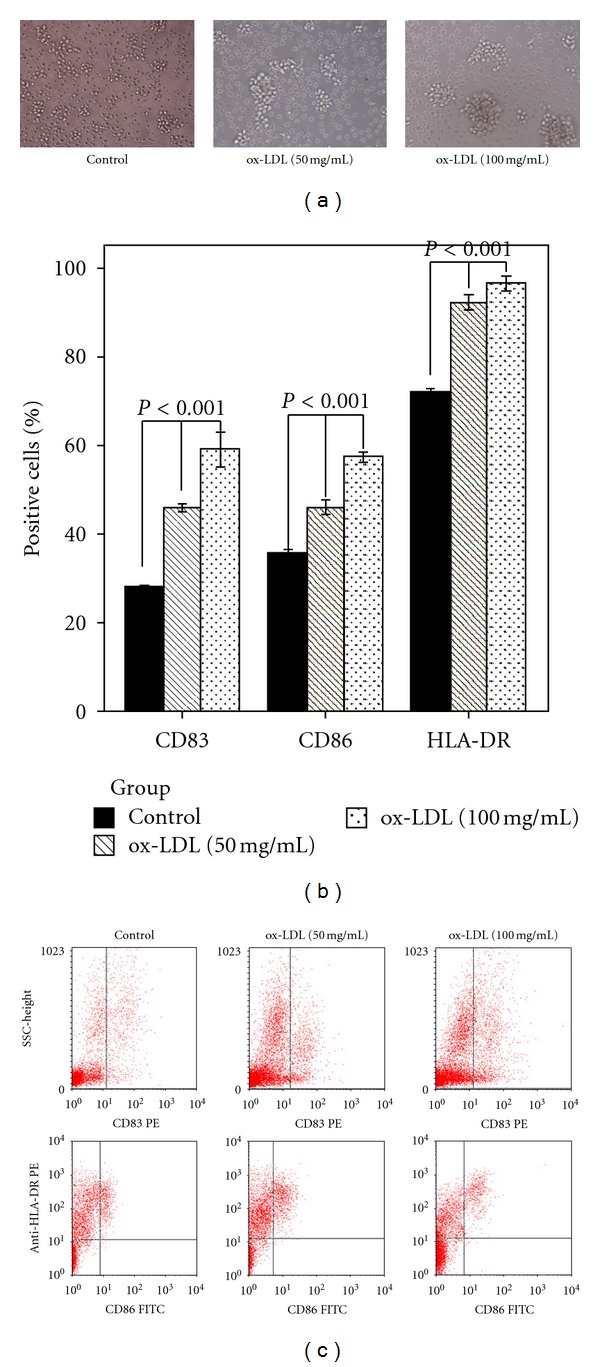
ox-LDL-induced morphological and phenotypic change in monocyte-derived DCs. Immature DCs were treated with different concentrations of ox-LDL in DC medium supplemented with 50 ng/mL GM-CSF (Peprotech) and 50 ng/mL IL-4 for 24 h. (a) DCs morphology were visualized by conventional light microscopy. (b) and (c) Cell surface molecules (CD83, CD86, and HLA-DR) were examined by FACS analysis. *P* < 0.001 compared among different concentration of ox-LDL-stimulated DCs.

**Figure 3:**
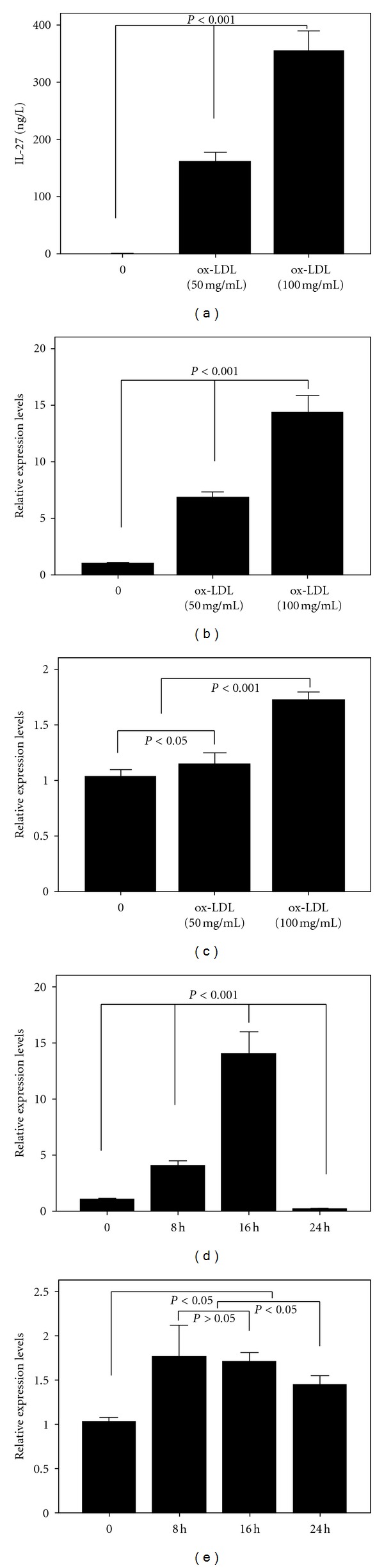
ox-LDL induced interleukin (IL)-27 production in monocyte-derived DCs. (a) Immature DCs were stimulated with different concentrations of ox-LDL for 24 h. The concentrations of IL-27 were analyzed using ELISA kit. (b) and (c) The mRNA expression levels of IL-27 p28 and ebi3 were evaluated by a real-time PCR analysis. The results are expressed as relative level to the control value. Results shown are means ± SD of six experiments. (d) and (e) Immature DCs were stimulated with ox-LDL (100 mg/mL) for 0, 8 h, 16 h, and 24 h. Then the mRNA expression levels of IL-27 p28 and ebi3 were evaluated by a real-time PCR analysis. The results are expressed as relative level to the control value. Results shown are means ± SD of six experiments.

ox-LDL has been shown to induce phenotypically mature DCs, with reduced uptake capacity, secreting IL-12 [22, 23]. Furthermore, IL-27, a new member of IL-12 family, also can be produced by activated dendritic cells, suggesting possible responsiveness to ox-LDL. To investigate a possible role for IL-27 in the proinflammatory effects of ox-LDL, we examined the capacity of ox-LDL to induce IL-27 production by DC. Human peripheral blood monocyte-derived DCs were stimulated with increasing concentrations of ox-LDL (0 to 100 mg/mL) and IL-27 expression was quantified by ELISA or its mRNA expression quantified by real-time PCR after 24 h incubation with ox-LDL. The results showed that ox-LDL upregulated expressions of CD83, CD86, and HLA-DR in DCs in a concentration-dependent manner (Figure 2). IL-27 protein (Figure 3(a)) and IL-27 p28 (Figure 3(b)) and ebi3 mRNA (Figure 3(c)) were expressed in a dose-dependent manner in response to ox-LDL. ox-LDL-induced IL-27 p28 mRNA expression peaked at 16 h (Figure 3(d)), while ebi3 was induced to a much lesser extent and peaked at 8 h (Figure 3(e)).

## 4. Discussion

CAD is a typically atherosclerotic disease. Recent evidence has indicated that inflammation and immunity, interacting with metabolic risk factors, are involved in mediating all stages of atherosclerosis, from low-density lipoprotein (LDL) cholesterol accumulation within the subendothelial space to atherosclerotic plaque progression, rupture, and thrombosis [24]. An intricate interplay between multiple immunological and biochemical mediators initiates and promotes these progresses.

IL-27 plays a dual role in regulating immune responses with both pro- and anti-inflammatory properties. It promotes the early Th1 differentiation via STAT1-mediated T-bet activation [25], but it suppresses the differentiation to Th2 [26, 27] and Th17 [28, 29] and production of proinflammatory cytokines [30, 31], and induces production of anti-inflammatory cytokines such as IL-10 by activated T cells [32].

 The role of IL-27 in promoting or suppressing inflammation may vary within different diseases. Currently available evidences show that IL-27 promotes the inflammation in diseases such as experimental crescentic glomerulonephritis [33], experimental hepatitis [34] and colitis [35], systemic sclerosis [36]; but suppresses the inflammation in diseases such as autoimmune arthritis [37], allergic asthma [38, 39], chronic inflammation of the central nervous system [29], experimental autoimmune encephalomyelitis [40–42], intraocular inflammation [43], Leishmania donovani infection [44]. IL-27 promotes early differentiation of TH1 and previous studies demonstrated marked elevations in the percentage of circulating Th1 cells in ACS patients [11]. In this study, we found that circulating levels of IL-27 are elevated in patients with CAD and positively correlated with the degree of the diseases, particularly in ACS, suggesting that IL-27 probably participates in the development of CAD. Further studies have shown that Th1 cells and their related cytokines promote the development and progression of atherosclerosis, whereas Th2 and regulatory T cells, and their related cytokines exert clear antiatherogenic activities [10, 11]. Furthermore, along with these findings, we found that circulating levels of IL-27 were significantly correlated with Genisini score which reflects the severity of coronary artery stenosis, indicating that IL-27 may promote the development and progression of CAD by inducing Th1 differentiation and related cytokine production.

A key role of DCs in bridging innate and adaptive immune systems has been recognized as antigen presentation since DCs were discovered [45, 46]. Upon acquiring “maturation/danger” signals, that is, ox-LDL, cytokines, and other molecules associated with inflammation or tissue damage, immature DCs rapidly undergo differentiation and maturation and migrate along chemotactic gradients to lymphatic tissues, where they form contacts with T cells to initiate a primary immune response [47]. In DC-T-cell contact, DCs secrete a spectrum of cytokines such as IL-12, IL-10, and IL-27, which determines their ability to polarize naive T cells into Th1, Th2, T regulatory cells (Treg), or Th17 cells [3, 4].

Several studies have demonstrated that mature DCs are upregulated and immature DCs are downregulated in patients with CAD [48–50], even monocytes-derived DCs from CAD patients showed more matured than from controls [48–50]. This study showed reduced counts of activated mDCs in CAD patients. This is possibly due to circulating DCs become activated, leaved the circulation, and migrated to lymphoid organs or to the site of inflammation (atherosclerotic plaques). However, if those activated DCs secrete IL-27 before or after leaving the circulation, the levels of this cytokine will still be elevated in spite of the lower count of activated circulating DCs [50]. ox-LDL can induce the maturation of DCs and activate them to produce cytokines such as IL-12 [22, 23]. Circulating ox-LDL is not only an independent predictor for CAD, but also an important atherogenic factor [51–53]. Consistent with previous studies, we observed evidence of a correlation between circulating ox-LDL and the severity of CAD [54]. In addition, we demonstrated that circulating ox-LDL is positively correlated with IL-27, which indicates that ox-LDL may be a major factor activating DCs to produce IL-27. To investigate this possibility, we studied the capacity of ox-LDL to induce IL-27 production by DC. The results showed that ox-LDL was a factor inducing secretion of IL-27 by DCs, suggesting an ox-LDL-induced and DC-mediated Th1-shift of the adaptive immunity.

Furthermore, IL-27 can be found in atherosclerotic plaques [55]. Expression of IL-27 may be closely related to DCs and ox-LDL in atherosclerotic plaques. Available evidence suggests that DCs play an important role in the pathogenesis of atherosclerosis. DCs are commonly found in the arterial intima [56] and have been shown to preferentially accumulate in regions predisposed to atherosclerosis in the normal murine aortic intima where they initiate nascent foam cell lesions at very early stages, as the atherosclerotic plaque starts to develop [57–59]. The uptake of ox-LDL by scavenger receptors leads to the accumulation of cholesterol within the foam cells of atherosclerotic lesions and have the effect of demonstrably altering DC function. In advanced atherosclerotic plaques, DCs are mostly present as a mature phenotype and accumulate preferentially within the vulnerable plaque shoulder by colocalizing with T cells [60, 61]. Several studies also showed that Th 1 cells play an important role in promoting the progression of atherosclerotic plaques [2, 10, 11]. The number of accumulated DCs is directly parallel to plaque complexity and inflammation [62, 63]. These reports showed that DCs accumulate in vulnerable plaque shoulders, and are related with plaque complexity are currently questioned and require confirmation or reevaluation [64]. Previous reports were based on a DC marker (fascin) that is expressed by neovascular endothelial cells that are prominently present in those conditions. Moreover, CD83 the marker of mature DCs [60, 63] is not DC-specific as it is expressed by lymphocytes and many other leukocytes [64]. Thus, combining the data from this and the previous studies leads to the assumption that in atherosclerotic plaques ox-LDL stimulate DCs to produce IL-27, which in turn further promotes Th1 differentiation. Thus, IL-27 potentially plays an important role in promoting the development and progression of atherosclerosis.

There are some limitations in our study. First, we did not observe the correlation between circulating IL-27, IFN-*γ*, IL-10, and circulating mature DC numbers. Second, we did not investigate whether ox-LDL can stimulate other antigen-presenting cells to produce IL-27, such as macrophages and B cells. Third, we did not directly investigate the effect of IL-27 on DCs in circulation and atherosclerotic plaques and the development of atherosclerosis *in vivo*. Answering these questions would have helped to better understand the role of IL-27 in patients with CAD.

In conclusion, we found that circulating levels of IL-27 are elevated and closely related to ox-LDL and the severity of coronary atherosclerotic lesions in patients with CAD. We herein provided the evidence that ox-LDL can stimulate DCs to produce IL-27. This study provides further understanding of the pathogenesis of atherosclerosis: elevated IL-27 might be one of the important cytokines that compose a regulatory network in immunity and inflammation to affect atherosclerosis. Further studies are required to elucidate whether IL-27 affects the development and progression of atherosclerosis, which may provide insights into novel therapeutic targets for controlling CAD.

## Figures and Tables

**Table 1:** Clinical characteristics of patient cohort.

	Control	SAP	UAP	AMI	*P*
Age (years)	63.793 ± 8.381	60.767 ± 10.553	62.232 ± 10.322	61.960 ± 9.583	0.700
Male gender, *n* (%)	21 (72.414)	21 (70)	39 (69.643)	32 (80)	0.865
Risk factors, *n* (%)					
Hypertension	9 (31.034)	9 (30)	20 (35.714)	11 (22)	0.493
Current smoking	12 (41.379)	16 (53.333)	25 (44.643)	20 (40)	0.691
Diabetes mellitus	5 (17.241)	7 (23.333)	12 (21.429)	13 (26)	0.836
Medication, *n* (%)					
Ca-antagonist	4 (13.793)	6 (20)	10 (17.857)	7 (14)	0.868
Aspirin	7 (24.138)	10 (30.303)	18 (32.143)	20 (40)	0.547
*β*-blockers	5 (17.241)	4 (13.333)	8 (14.286)	6 (12)	0.933
Other antiplatelet agents	2 (6.897)	3 (10)	7 (12.5)	12 (24)	0.132
TC (mmol/L)	5.188 ± 1.271	4.807 ± 1.196	4.875 ± 1.229	4.885 ± 1.166	0.618
LDL-C (mmol/L)	2.387 ± 0.669	2.918 ± 1.000^a^	2.798 ± 0.682^a^	2.916 ± 0.871^a^	0.029
TG (mmol/L)	1.292 ± 0.636	1.311 ± 0.470	1.338 ± 0.528	1.398 ± 0.544	0.830
Leukocytes (G/L)	7.060 ± 1.491	7.664 ± 2.031	7.193 ± 1.877	7.138 ± 2.042	0.587
Creatinine (*μ*mol/L)	89.931 ± 20.664	98.100 ± 19.744	95.750 ± 16.282	94.620 ± 18.951	0.380
Uric acid (*μ*mol/L)	283.830 ± 95.354	324.730 ± 111.372	312.930 ± 108.287	334.500 ± 75.393^a^	0.161
cTnI (*μ*g/L)	0.012 ± 0.004	0.017 ± 0.010^a^	0.054 ± 0.112^a^	15.572 ± 16.487^a, b, c^	<0.001

Values are expressed as percentages or mean ± SD.

^a^ *P* < 0.05 versus control subjects; ^b^ *P* < 0.05 versus stable angina pectoris patients; ^c^ *P* < 0.05 versus unstable angina pectoris patients.

SAP: stable angina pectoris; UAP: unstable angina pectoris; AMI: acute myocardial infarction; TC: total cholesterol; LDL-C: low-density lipoprotein cholesterol; TG: triglycerides; cTnI: cardiac troponin I.

**Table 2:** Circulating IL-27, ox-LDL, and Genisi score of subjects in each group.

	Control	SAP	UAP	AMI	*P*
ox-LDL (*μ*g/L)	212.310 ± 48.990	356.810 ± 84.867^a^	539.240 ± 95.205^a, b^	629.050 ± 149.541^a, b, c^	<0.001
IL-27 (ng/L)	0.467 ± 0.235	1.020 ± 0.383^a^	1.401 ± 0.389^a, b^	1.444 ± 0.336^a, b^	<0.001
Genisin score	2.000 ± 2.891	39.350 ± 28.708	62.250 ± 36.538	85.080 ± 36.591^a, b,c^	<0.001

Values are expressed as mean ± SD.

^a^ *P* < 0.05 versus control subjects; ^b^ *P* < 0.05 versus stable angina pectoris patients; ^c^ *P* < 0.05 versus unstable angina pectoris patients.

SAP: stable angina pectoris; UAP: unstable angina pectoris; AMI: acute myocardial infarction.
